# Inspired by Nature: Antioxidants and Nanotechnology

**DOI:** 10.3390/antiox7080101

**Published:** 2018-07-29

**Authors:** Claus Jacob

**Affiliations:** Division of Bioorganic Chemistry, School of Pharmacy, Saarland University, Campus B 2.1, Saarbruecken D-66123, Germany; c.jacob@mx.uni-saarland.de; Tel.: +49-681-302-3129; Fax: +49-681-302-3464

Since the advent of modern nanotechnology a couple of decades ago, the field of “nano-bio-med” has attracted particular interest, culminating in an almost meteoric rise of common, feasible, more speculative, and, on occasion, outrightly exotic applications of nanomaterials. Intriguingly, whilst many of these nanomaterials are the products of highly sophisticated and eloquent manufacturing methods, others are entirely natural and older than humankind. Indeed, as this Special Issue illustrates, natural nanomaterials encompass a wide range of diverse substances which provide a fertile ground for inspiration, innovation, research and development.

On the one side, nanoscopic particles are formed by natural processes, such as abrasion, precipitation, combustion and bioreduction [1]. Nanoscopic minerals in water, deposits of elemental sulfur at and near mineral wells, soot particles emanating from volcanos and elemental sulfur, selenium and even tellurium particles produced by bacteria, such as certain *Lactobacilli*, are just some of the better-known examples of such “natural” nanocomposites [2,3,4,5,6,7]. These natural nanoparticles differ from human-made nanoparticles based on natural materials, which can be generated by different techniques, most obviously by nanosizing macroscopic and microscopic substances [8]. The manuscript by Sarfraz et al. describes the application of such a sequence of grinding, milling, stirring and high-pressure homogenization of entire plant parts for the production of biologically active nanoparticles derived from the African Mistletoe (*Loranthus micranthus*) [9]. Such nanosized particles are traditionally kept in a nanosuspension stabilized by an appropriate surfactant, which somewhat limits their practical applications. The manuscript by Griffin et al. therefore considers a succession of nanosizing, lyophilization and resuspension (“NaLyRe”) as a possible approach to circumvent some of these drawbacks [10].

Besides nanosizing entire plants or plant parts, alternative and perhaps “cleaner” approaches to nanomaterials of natural products consider the incorporation of active natural compounds, such as chemically pure antioxidants, into defined “carrier” nanoparticles. Here, Umerska et al. show that polymeric nanoparticles based on Eudragit^®^ RLPO and loaded with curcumin can be produced and easily resuspended after freeze drying. They also provide good release properties when compared to particles based on polylactic-*co*-glycolic acid or polycaprolactone [11].

These materials are “natural” as far as the composition of the material is concerned, even though the method of “nanosizing” is not necessarily so. In the future, “twice natural” materials based on natural products and formed by natural processes may become of special interest. Such materials are feasible, as the example of nano-deposits in bacteria and fungi highlights, although they are rarer in practice and probably would need to be separated from larger particles and purified by adequate separation methods. Chalcogen nanoparticles produced by bacteria such as *Staphylococcus carnosus* and by certain yeasts, such as *Saccharomyces cerevisiae* and *Candida glabrata*, are also not solely “inorganic”, as one may suspect initially, but are often coated with proteins and enzymes, which renders these nicely shaped natural nanorods and nanospheres particularly interesting [1,2,12,13,14,15,16].

Regardless of origin, natural nanomaterials are rather special once their biological activity and possible applications in Biology, Medicine, Pharmacy and even in Agriculture are considered. Whilst macroscopic particles usually lack any pronounced biological activity due to low bioavailability, and rapidly and completely soluble materials act almost exclusively via chemical interactions, nanomaterials exert their activity on biological systems via more than one mechanism. These materials are (a) generally reasonably bioavailable thanks to their small size, which enables them to form nanosuspensions and to cross biological barriers, (b) highly reactive chemically as a result of their large surface-to-volume ratio, (c) excellent release systems for biologically active molecules, and (d) also able to act via physical rather than chemical interactions, for instance by disrupting cellular and subcellular structures. The examples discussed in the manuscripts of this Special Issue bear witness to this specific cocktail of actions and interactions. They range from antioxidant and cytotoxic activities of various natural products and substances to the delivery of reduced glutathione (GSH) and a kind of “nano-therapy” involving reactive oxygen species (ROS). Certain nanomaterials may protect cells by releasing GSH, as discussed by Gaucher et al. [17]. Others may turn out to be toxic, and the manuscript by Brenneisen and Reichert discusses a novel perspective on this particular matter of (nano-)toxicity [18].

Indeed, nanosized materials may be rather disruptive, and nanotoxicity, often associated with the formation of ROS, may not only be detrimental, but from a therapeutic perspective, may also open up new and innovative avenues in the design of agents acting via a combination of physical and chemical modes of action [19,20].

In this, and in various other respects, the manuscripts in this Special Issue foretell a variety of natural or artificially generated nanomaterials which differ dramatically from their macroscopic and soluble counterparts and may provide a range of interesting leads for innovative food supplements, therapeutics, cosmetics and phyto-protectants. So indeed, size matters, and apart from being beautiful, small may also—literally—be quite fruitful in natural product research and development.
